# Voluntary exercise prevents abnormal muscle mitochondrial morphology in cancer cachexia mice

**DOI:** 10.14814/phy2.15016

**Published:** 2021-08-24

**Authors:** Yu Kitaoka, Mitsunori Miyazaki, Shin Kikuchi

**Affiliations:** ^1^ Department of Human Sciences Kanagawa University Yokohama Japan; ^2^ Department of Integrative Physiology Graduate School of Biomedical and Health Sciences Hiroshima University Hiroshima Japan; ^3^ Department of Physical Therapy School of Rehabilitation Sciences Health Sciences University of Hokkaido Ishikari‐Tobetsu Japan; ^4^ Department of Anatomy 1 Sapporo Medical University School of Medicine Sapporo Japan

**Keywords:** cancer cachexia, mitochondria, oxidative stress, skeletal muscle, voluntary exercise

## Abstract

This study aimed to examine the effects of voluntary wheel running on cancer cachexia‐induced mitochondrial alterations in mouse skeletal muscle. Mice bearing colon 26 adenocarcinoma (C26) were used as a model of cancer cachexia. C26 mice showed a lower gastrocnemius and plantaris muscle weight, but 4 weeks of voluntary exercise rescued these changes. Further, voluntary exercise attenuated observed declines in the levels of oxidative phosphorylation proteins and activities of citrate synthase and cytochrome *c* oxidase in the skeletal muscle of C26 mice. Among mitochondrial morphology regulatory proteins, mitofusin 2 (Mfn2) and dynamin‐related protein 1 (Drp1) were decreased in the skeletal muscle of C26 mice, but exercise resulted in similar improvements as seen in markers of mitochondrial content. In isolated mitochondria, 4‐hydroxynonenal and protein carbonyls were elevated in C26 mice, but exercise blunted the increases in these markers of oxidative stress. In addition, electron microscopy revealed that exercise alleviated the observed increase in the percentage of damaged mitochondria in C26 mice. These results suggest that voluntary exercise effectively counteracts mitochondrial dysfunction to mitigate muscle loss in cachexia.

## Graphical abstract

This study examined the effects of voluntary exercise on cancer cachexia‐induced mitochondrial alterations in mouse skeletal muscle. We found that exercise effectively counteracts mitochondrial dysfunction to mitigate muscle loss in cachexia.

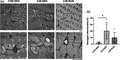

## 1 INTRODUCTION

Cancer cachexia is a systemic syndrome that is characterized by the loss of body weight, skeletal muscle wasting, and metabolic disorders (Fearon et al., 2012). Skeletal muscle mass or strength is considered a predictor of all‐cause mortality in older adults (Metter et al., 2002; Srikanthan & Karlamangla, 2014). It has been demonstrated that mitochondrial dysfunction in skeletal muscle contributes to reduced muscle weight in various animal models, including physical inactivity, denervation, and aging (Peterson et al., 2012; Powers et al., 2012). Evidence involving mitochondrial impairments in cancer cachexia has also been reported (Tzika et al., 2013; VanderVeen, Fix, Carson, 2017). Considering that mitochondrial degeneration precedes skeletal muscle loss in cancer cachexia (Brown et al., 2017), maintenance of mitochondrial homeostasis may contribute to the prevention of cachexia‐induced muscle wasting.

Mitochondria are multifunctional organelles that continuously alter their shape through fission and fusion to maintain quality and function (Picard et al., 2013). Previous studies using muscle‐specific knockout of mitofusin 1 (Mfn1) and Mfn2, which are involved in mitochondrial fusion (Chen et al., 2010), and dynamin‐related protein 1 (Drp1), which is involved in mitochondrial fission (Dulac et al., 2020), showed severe muscle atrophy in both animal models, suggesting that mitochondrial dynamics are important not only for efficient energy production but also for the maintenance of skeletal muscle mass. We have previously reported that chronic electrical stimulation mimicking resistance exercise induces muscle hypertrophy and increases mitochondrial fusion regulatory proteins (Kitaoka, Nakazato, et al., 2016; Kitaoka, Ogasawara, et al., 2015), whereas mitochondrial fission regulatory proteins were increased (but fusion regulatory proteins were decreased) under conditions that lead to muscle atrophy such as denervation (Kitaoka, Takeda, et al., 2016). Previous studies using a genetic model of colon cancer (White et al., 2012) or elderly patients with gastric cancer (Marzetti et al., 2017) have demonstrated that the abnormal expression of proteins regulating mitochondrial dynamics in skeletal muscle was induced with the progression of cachexia.

Maintaining regular physical activity results in multiple adaptations in skeletal muscle, including mitochondrial biogenesis, which leads to an improved healthspan (Cartee et al., 2016; Zampieri et al., 2015). Physical exercise also inhibits tumor growth and contributes to improved muscle mass in rodents (Deuster et al., 1985; Pedersen et al., 2015). While much work has been done on the effects of exercise on skeletal muscle functional properties in tumor‐bearing mice (Ranjbar et al., 2019; Vanderveen, Fix, Counts, et al., 2020), it remains unknown whether exercise prevents abnormal muscle mitochondrial morphology. In this study, we examined the hypothesis that physical exercise alleviates mitochondrial dysfunction and contributes to the maintenance of skeletal muscle mass in a mouse model of cancer cachexia. Considering the potential risk of muscle damage associated with forced exercise using a treadmill (Fraysse et al., 2004; Okano et al., 2005), we employed voluntary running exercise which is expected to improve skeletal muscle function without excessive exacerbation of muscle damage (Baltgalvis et al., 2012; Hayes & Williams, 1996).

## 2 MATERIALS AND METHODS

### 2.1 Animal care and use

All experimental procedures performed in this study were conducted in accordance with the institutional guidelines provided for the care and use of laboratory animals, which were approved by the Animal Ethics and Research Committee of the Health Sciences University of Hokkaido (No. 20‐006). Animals were housed in a temperature‐controlled and humidity‐controlled room (24 ± 1℃ and 50%–60%) and maintained on a 12‐h light/12‐h dark cycle with access to food and water *ad libitum*. Male CD2F1/Slc mice (Japan SLC) aged between 7 and 8 weeks were used in this study. Colon 26 (C26) carcinoma tumor‐bearing CD2F1 mice were employed as the model for the study of cancer cachexia. Mice were randomly assigned to the control sedentary (CON‐SED), C26‐bearing sedentary (C26‐SED), and C26‐bearing voluntary running (C26‐RUN) groups (*n* = 9 for each group). The C26 carcinoma (RCB2657) was provided by RIKEN BRC through the National Bio‐Resource Project of MEXT, Japan. All cell culture experiments were performed in a humidified environment at 37℃ in a 5% CO_2_ atmosphere. The C26 cells were grown in RPMI‐1640 medium (FUJIFILM Wako Pure Chemical Corporation) supplemented with 10% fetal bovine serum and penicillin–streptomycin. After trypsinization and neutralization, 1 × 10^6^ C26 cells per individual were suspended with 100 μl of phosphate‐buffered saline (PBS) and subcutaneously implanted into the flank region of the abdominal wall unilaterally using a 26G syringe. The control group was injected with an equal amount of PBS. All mice were kept in individual cages with free‐spinning running wheels (SN‐450; Shinano), and access to a running wheel was allowed to the C26‐RUN group immediately after tumor‐bearing. Non‐exercise animals (CON‐SED and C26‐SED) were kept in the same cages with the entrance to the wheel sealed off. Body weight, muscle strength, and dietary intake were measured over time from 1 to 4 weeks following C26 cell transplantation while confirming tumor tissue formation. Forelimb grip strength was measured using a grip strength meter (MK‐380M; Muromachi Kikai), and the average of three trials was employed as the value for each individual. At the end of each experimental period (4 weeks following tumor‐bearing), the mice were anesthetized, and the muscle samples were excised, weighed, quickly frozen in liquid nitrogen, and stored at −80℃. After the completion of experimental treatments, the mice were euthanized by cervical dislocation under anesthesia.

### 2.2 Whole muscle lysate

Gastrocnemius muscles were lysed in an ice‐cold homogenization buffer (1% NP‐40, 0.5% sodium deoxycholate, 0.1% sodium dodecyl sulfate [SDS], 50 mM NaCl, 20 mM Tris–HCl [pH, 7.6], 1 mM phenylmethylsulfonyl fluoride, 5 mM benzamidine, 1 mM ethylenediaminetetraacetic acid (EDTA), 5 mM *N*‐ethylmaleimide, 50 mM NaF, 25 mM B‐glycerophosphate, 1 mM sodium orthovanadate, and 1× protease inhibitor cocktail [Nacalai Tesque]). The lysed samples were then centrifuged at 16,000 *g* for 10 min at 4℃, and the supernatants were collected for analysis. The protein concentration was determined using a BCA protein assay kit (Thermo Fisher Scientific).

### 2.3 Mitochondrial isolation

Mitochondrial fractions were isolated using differential centrifugation as previously described (Wakabayashi et al., 2020). Briefly, gastrocnemius muscles were well minced using scissors in buffer A (PBS and 10 mM EDTA, pH 7.4) and then incubated with 0.025% trypsin (209–16941; FUJIFILM Wako Pure Chemical Corporation) for 5 min on ice. After centrifugation at 200 *g* for 5 min at 4℃, the pellets were resuspended in buffer B (50 mM 3‐(*N*‐morpholino)propanesulfonic acid [MOPS], 100 mM KCI, 1 mM ethylene glycol tetraacetic acid [EGTA], 5 mM MgSO_4_, and 2.0 g/L bovine serum albumin, pH 7.1) and homogenized using a Potter glass homogenizer. Following centrifugation at 500 *g* for 10 min at 4℃, the collected supernatants were centrifuged at 10,000 *g* for 10 min at 4℃. The mitochondrial pellets were then washed in buffer C (50 mM MOPS, 100 mM KCI, 1 mM EGTA, and 5 mM MgSO_4_) and resuspended in radioimmunoprecipitation assay buffer. After the isolation procedure, the total protein content of the samples was determined using a BCA protein assay kit (Thermo Fisher Scientific). Mitochondrial samples were used for analyses of 4‐hydroxynonenal (4‐HNE) and protein carbonyl contents.

### 2.4 Western blotting

Equal amounts of protein were loaded onto 10%–12% SDS‐polyacrylamide gel electrophoresis gels and separated by electrophoresis. Proteins were transferred to polyvinylidene difluoride (PVDF) membranes, and western blotting was performed using primary antibodies 4‐HNE (ab48506), total OXPHOS Rodent WB Antibody Cocktail (ab110413), Fis1 (ab96764), Drp1 (ab56788), and Mfn2 (ab124773) from Abcam and Opa1 (#612606) from BD Transduction Laboratories. Ponceau staining was used to verify consistent loading. Blots were scanned and quantified using a C‐Digit Blot Scanner (LI‐COR).

### 2.5 Protein carbonyl content

Protein carbonyl content was measured using a commercially available kit (#ROIK03; SHIMA Laboratories). After mitochondrial proteins were transferred to a PVDF membrane, the membrane reacted with dinitrophenylhydrazine, followed by western blotting.

### 2.6 Enzyme activity

Plantaris muscles were homogenized in 50 (v/w) of 100 mmol/L of potassium phosphate buffer. Maximal activities of citrate synthase (CS) and cytochrome *c* oxidase (COX) were measured spectrophotometrically following established protocols (Spinazzi et al., 2012).

### 2.7 Transmission electron microscopy imaging

Additional mice were prepared (*n* = 4–5 for each group), and gastrocnemius muscles were fixed with 2% glutaraldehyde and 2% paraformaldehyde in 0.1 M cacodylate buffer (pH 7.4) for 24 h at 4℃ after cardiac perfusion of the fixative. The muscle was cut into ~1 mm^3^ blocks and then washed in 0.1 M cacodylate buffer three times for 5 min. Next, the muscles were post‐fixed with 1% osmium tetroxide in 0.1 M cacodylate buffer for 1.5 h at room temperature (RT) with gentle shaking and then dehydrated through a graded ethanol series at RT. After dehydration, the muscles were infiltrated with a graded mixture of acetone and epoxy resin at RT and then embedded in 100% epoxy resin at 60℃ for 48 h. Afterward, 1‐μm thick sections were made to check the orientation of the embedded bundle and section quality. Ultrathin sections were cut using a diamond knife on an ultramicrotome, and the sections were mounted on grids. These ultrathin sections were stained with uranyl acetate and lead citrate at RT for 20 and 5 min, respectively. Finally, a transmission electron microscope (JEM‐1400; JEOL Ltd.) was used to obtain images as previously reported (Ando et al., 2021). A grid‐sampling method was used to determine the percentage of damaged mitochondria present in the interfibrillar region. Briefly, a square grid was placed over the image at 20,000× magnification, and all mitochondria of normal and damaged morphology located in the region were counted. We defined damaged mitochondria as mitochondria with swelling and/or incomplete formation of cristae structure. Nonoverlapped areas were randomly selected and identically distributed in each electron microscopy image such that a total of 200 mitochondria per muscle sample on average was analyzed.

### 2.8 Statistical analysis

All data were expressed as mean ± standard deviation. One‐way analysis of variance (ANOVA) was used to assess the significance of differences. When ANOVA revealed significant differences, Fisher’s protected least significant difference post hoc tests were used to compare the experimental groups. All statistical analyses were performed using GraphPad Prism 9 (GraphPad Software). Statistical significance was defined as *p* < 0.05.

## 3 RESULTS

### 3.1 Voluntary exercise attenuates the loss of body weight and skeletal muscle mass associated with cancer cachexia

**FIGURE 1:**
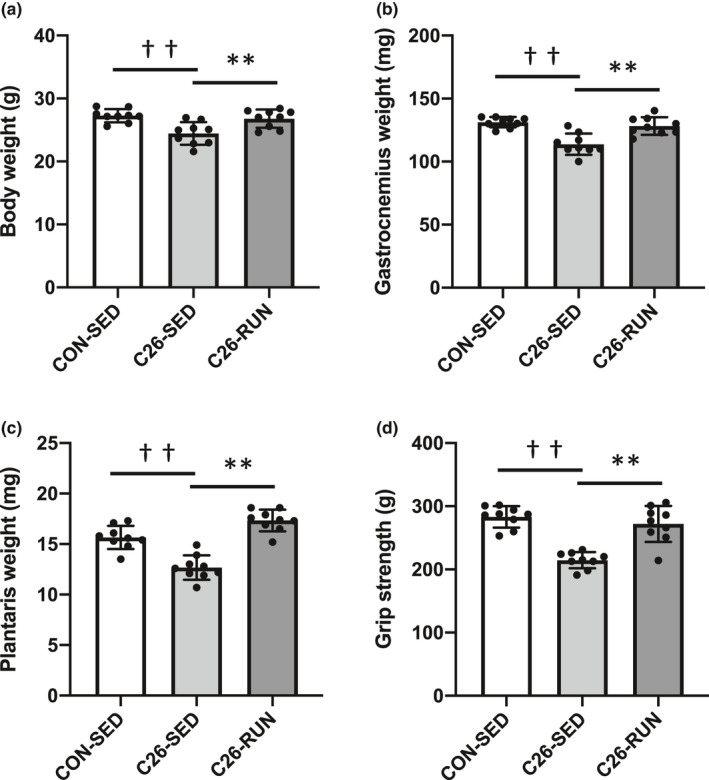
Body weight, skeletal muscle mass, and grip strength. (a) tumor‐free body weight, (b) gastrocnemius muscle weight, (c) plantaris muscle weight, (d) forelimb grip strength. Values are expressed as mean ± standard deviation; *n* = 9 in each group. ^**^ *p* < 0.01, significant difference between C26‐SED and C26‐RUN. ^††^ *p* < 0.01, significant difference between CON‐SED and C26‐SED

The physical characteristics of the mice in each group are shown in Figure 1. The development of cancer cachexia was observed in the C26‐SED group, including significant weight loss (Figure 1a), decreased skeletal muscle mass (Figure 1b,c), and weakened grip strength (Figure 1d), compared with CON‐SED mice. The deterioration of the physical characteristics associated with C26 tumor‐bearing was successfully improved in C26‐RUN mice, indicating a preventive effect of voluntary exercise on the development of cancer cachexia. We confirmed that the daily running distances in C26‐RUN mice averaged 13.1 ± 1.0 km/day. Detailed time course data of running distance (Figure S1a), food intake (Figure S1b), and grip strength (Figure S1c) over 4 weeks are provided in Figure S1. Of importance, the tumor weight of C26‐RUN mice was lower than CON‐SED (Figure S1d), suggesting that physical exercise has an inhibitory effect on tumor growth, as in previous studies (Deuster et al., 1985; Pedersen et al., 2015).

### 3.2 Voluntary exercise attenuates mitochondrial abnormality in skeletal muscle induced by cancer cachexia

**FIGURE 2:**
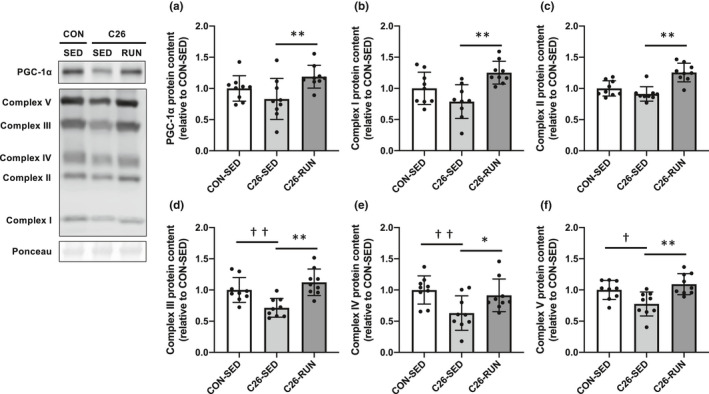
Content of mitochondria‐related proteins in skeletal muscle. (a) PGC‐1α, (b) complex I, (c) complex II, (d) complex III, (e) complex IV, (f) complex V protein levels. Values are expressed as mean ± standard deviation; *n* = 9 in each group. ^*^ *p* < 0.05, ^**^ *p* < 0.01, significant difference between C26‐SED and C26‐RUN. ^†^ *p* < 0.05, ^††^ *p* < 0.01, significant difference between CON‐SED and C26‐SED

**FIGURE 3:**
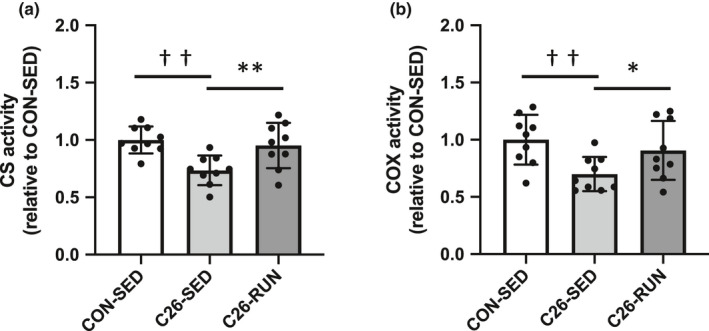
Mitochondrial enzyme activity. (a) CS, (b) COX activity. Values are expressed as mean ± standard deviation; *n* = 9 in each group. ^*^ *p* < 0.05, ^**^ *p* < 0.01, significant difference between C26‐SED and C26‐RUN. ^††^ *p* < 0.01, significant difference between CON‐SED and C26‐SED. COX, cytochrome *c* oxidase; CS, citrate synthase

**FIGURE 4:**
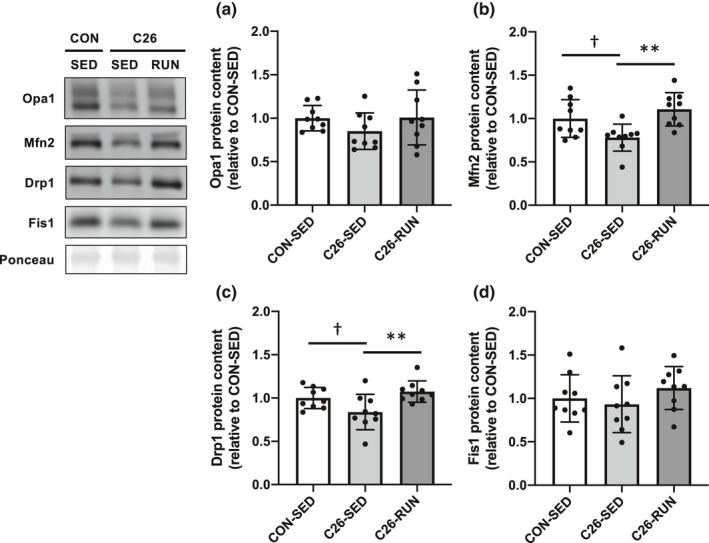
Mitochondrial fusion and fisson regulatory proteins. (a) Opa1, (b) Mfn2, (c) Drp1, (d) Fis1 protein levels. Values are expressed as mean ± standard deviation; *n* = 9 in each group. ^**^ *p* < 0.01, significant difference between C26‐SED and C26‐RUN. ^†^ *p* < 0.05, significant difference between CON‐SED and C26‐SED. Drp1, dynamin‐related protein 1; Mfn2, mitofusin 2

**FIGURE 5:**
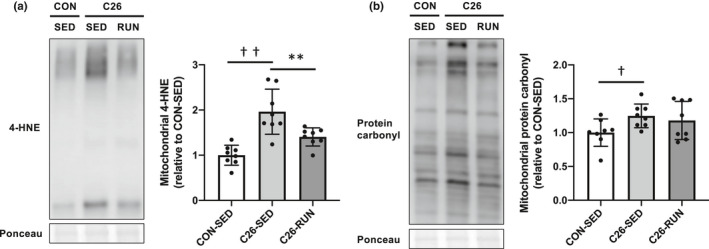
Mitochondrial oxidative stress. (a) 4‐HNE, (b) protein carbonyls in mitochondrial fractions. Values are expressed as mean ± standard deviation; *n* = 8 in each group. ^**^ *p* < 0.01, significant difference between C26‐SED and C26‐RUN. ^†^ *p* < 0.05, ^††^ *p* < 0.01, significant difference between CON‐SED and C26‐SED. 4‐HNE, 4‐hydroxynonenal

**FIGURE 6:**
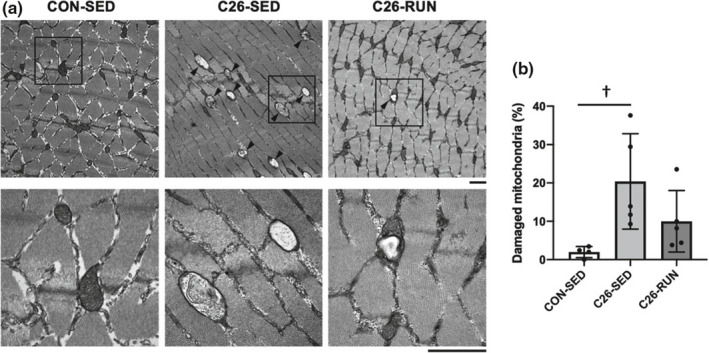
Transmission electron microscopy analysis of mitochondria. (a) Representative images (Scale bars: 1 μm). Arrowheads indicate damaged mitochondria with swelling and/or incomplete formation of cristae structure. (b) Percentage of damaged mitochondria. Values are expressed as mean ± standard deviation; *n* = 4–5 in each group. From each mouse on average, more than 200 mitochondria were examined. ^†^ *p* < 0.05, significant difference between CON‐SED and C26‐SED

As the cancer cachexia developed, the expression levels of OXPHOS subunit proteins (complex III, IV, and V) in skeletal muscle were significantly decreased, while all OXPHOS subunit protein levels were upregulated following 4 weeks of voluntary exercise compared to the C26‐SED mice. The expression of PGC‐1α, a master regulator of mitochondrial biogenesis, was also enhanced in response to exercise intervention (Figure 2). Along with the protein expression levels shown by Western blotting, the enzymatic activities of CS (representatives of the tricarboxylic acid cycle) and COX (representatives of the electron transport chain), which are indicators of mitochondrial content, were also decreased with cancer cachexia (Figure 3). The expression levels of Drp1 and Mfn2, regulatory proteins of mitochondrial dynamics, were also decreased (Figure 4). In addition, markers of oxidative stress including 4‐HNE and protein carbonyls were markedly elevated in isolated mitochondria (Figure 5), suggesting that mitochondrial abnormality in skeletal muscle is accompanied by cancer cachexia. The observations showing abnormal regulation of mitochondrial quantity were rescued with the intervention of voluntary exercise (Figures 2, 3, 4, 5). We also examined the morphological alterations of mitochondria in skeletal muscle using electron microscopy and found that physical exercise could alleviate an increased appearance of damaged mitochondria with disrupted cristae structure in cancer cachexia mice (Figure 6).

## 4 DISCUSSION

The major question to address in this study was whether physical exercise in C26 tumor‐bearing mice prevents skeletal muscle mitochondrial abnormality induced by cancer cachexia. According to our results, decreased level of OXPHOS subunit proteins (complex III, IV, and V), mitochondrial content (determined by enzymatic activity), and regulatory proteins of mitochondrial dynamics (Drp1 and Mfn2), were corrected by the voluntary exercise intervention, thereby suggesting that physical exercise is an effective therapeutic strategy to reduce mitochondrial abnormality associated with cancer cachexia.

Reduced mitochondrial quality and content in skeletal muscle are implicated in several pathological conditions, including age‐related (Chabi et al., 2008) and disuse‐induced (Min et al., 2011) muscle atrophy, muscular dystrophy (Kuznetsov et al., 1998), and type 2 diabetes (Goodpaster, 2013). Consistent with previous studies that reported reductions in mitochondrial enzyme activities with cancer cachexia (Antunes et al., 2014; Fermoselle et al., 2013), we found significant reductions in mitochondrial OXPHOS proteins, as well as CS and COX activities, in skeletal muscle of C26 tumor‐bearing mice. Furthermore, previous reports of muscle proteomic profiling have revealed that regulatory proteins of oxidative metabolism were downregulated in C26 mice (Barreto et al., 2016; Shum et al., 2018), suggesting that C26‐induced cancer cachexia decreases mitochondrial content in skeletal muscle. Although we did not measure mitochondrial respiratory function in the current study, evidence supports the notion that cancer cachexia induces muscle mitochondrial dysfunction. The Yoshida AH‐130 tumor model leads to a marked decrease in muscle ATP content (Fontes‐Oliveira et al., 2013), whereas the expression of uncoupling proteins was upregulated (Sanchís et al., 1998). Similarly, the ATP synthesis rate was reduced while mitochondrial uncoupling occurred in Lewis lung carcinoma (LLC) models of cancer cachexia (Tzika et al., 2013). Furthermore, oxygen consumption was decreased in mitochondria isolated from the skeletal muscle of cachectic rats with peritoneal carcinosis (Julienne et al., 2012) and mice bearing the LP07 lung tumor (Fermoselle et al., 2013). Recent studies demonstrated that mitochondrial respiratory capacity was impaired by C26‐induced cancer cachexia in saponin‐permeabilized soleus (Neyroud et al., 2019) and gastrocnemius (Halle et al., 2019) myofibers. Importantly, these changes were independent of mitochondrial volume density.

Although the mechanism by which cancer cachexia causes mitochondrial dysfunction in skeletal muscle is still unclear, we hypothesized that it is due to alterations in mitochondrial fusion and fission as the maintenance of mitochondrial morphology is critical for normal cellular function (Hood et al., 2019). In this study, we found that muscle Drp1 and Mfn2 protein levels were reduced in the skeletal muscle of C26 mice. During the development of cachexia in Apc^Min/+^ mice, which is a genetic model of colon cancer, protein expression of Mfn2 was decreased in skeletal muscle (Brown et al., 2017; White et al., 2012). Our results were consistent with recent reports in C26 mice showing reduced expression of mediators of mitochondrial fission and fusion (Ballarò et al., 2019; Barreto et al., 2016). To determine whether impaired mitochondrial dynamics lead to elevated mitochondrial oxidative damage, we measured the oxidative modifications of proteins and lipids in isolated mitochondrial fractions. Our observations of the increase in 4‐HNE and protein carbonyls suggest that cancer cachexia‐induced oxidative stress leads to mitochondrial toxicity. Indeed, we confirmed the increased proportion of damaged mitochondria in skeletal muscle of C26 mice using transmission electron microscopy.

A growing body of evidence shows that exercise can be an effective intervention for counteracting cancer cachexia using rodent models such as AH‐130 (Tanaka et al., 2019) and C26 (Khamoui et al., 2016). Because exercise is a powerful inducer of mitochondrial adaptations in skeletal muscle (Egan & Zierath, 2013), we examined whether voluntary wheel running prevents abnormal muscle mitochondrial morphology in C26 mice. In this study, we found that exercise rescued the decrease in markers of mitochondrial content and dynamics in skeletal muscle of C26 mice. Moreover, isolated mitochondria from skeletal muscle demonstrated that exercise normalized the markers of oxidative damage in C26 mice, indicating the efficiency of voluntary exercise on cancer cachexia‐induced mitochondrial dysfunction. Importantly, observations using transmission electron microscopy also showed that exercise prevented mitochondrial morphological abnormalities in the skeletal muscle of C26 mice.

This rescue of mitochondrial abnormalities may be due to exercise‐induced PGC‐1α, which is a key regulator of mitochondrial homeostasis. Interestingly, muscle‐specific PGC‐1α overexpression was reported to prevent LLC‐induced muscle atrophy (Pin et al., 2015), while there is a conflicting report regarding the role of PGC‐1α (Wang et al., 2012). Similarly, treatment with AICAR (Pigna et al., 2016) or trimetazidine (Molinari et al., 2017), which are considered “exercise mimetics” that upregulate PGC‐1α, was shown to partially restore muscle mass and myofiber cross‐sectional area in C26 mice, suggesting that the induction of PGC‐1α is crucial for exercise‐induced alleviation of muscle mitochondrial dysfunction in cancer cachexia. However, at present, the causal role of mitochondria in cancer cachexia‐induced muscle atrophy remains unclear. A recent study demonstrated that the administration of the mitochondria‐targeted peptide SS‐31 prevented muscle dysfunction in C26 mice (Smuder et al., 2020), indicating that mitochondrial dysfunction is an underlying factor that contributes to the pathology of cancer cachexia.

## 5 CONCLUSION

In this study, we showed that exercise attenuates the decline in the levels of markers of mitochondrial content and dynamics, thus preventing an accumulation of mitochondrial oxidative damage, and subsequently, abnormal mitochondrial morphology in the skeletal muscle of C26 mice. Our results also suggest that voluntary exercise is a simple yet effective therapeutic strategy to mitigate muscle loss in cachexia by counteracting mitochondrial dysfunction.

## CONFLICT OF INTERESTS

The authors have no conflicts to declare.

## AUTHOR CONTRIBUTIONS

Yu Kitaoka and Mitsunori Miyazaki conceived and designed the project; Yu Kitaoka, Mitsunori Miyazaki, and Shin Kikuchi acquired, analyzed, and interpreted the data; Yu Kitaoka and Mitsunori Miyazaki wrote and revised the manuscript; Mitsunori Miyazaki edited the paper; Mitsunori Miyazaki directed and managed the whole project.

## DATA AVAILABILITY STATEMENT

The data that support the findings of this study are available from the corresponding author upon reasonable request from a qualified researcher.

## Supporting information



Figure S1.
