# Supplementary material for: Voluntary exercise prevents abnormal muscle mitochondrial morphology in cancer cachexia mice
Source: Physiol Rep. 2021 Aug 24;9(16):e15016. doi: 10.14814/phy2.15016 (PMC8383714; doi:10.14814/phy2.15016)
Supplement: Supplementary file 1 — Figure S1. [file PHY2-9-e15016-s001.pdf]

Supplemental Figure 1.

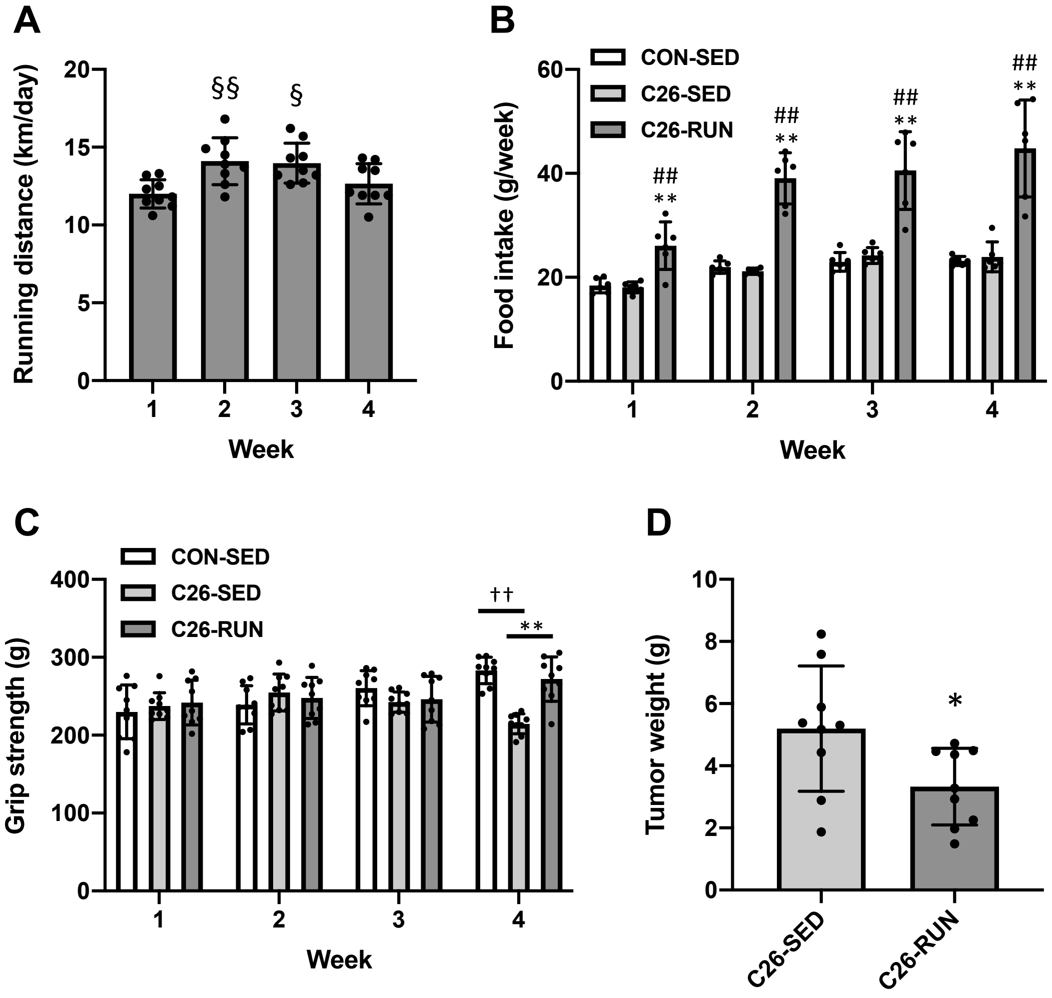

Supplemental Figure 1. Treatment validation data. (A) daily running distance for each week (n = 9 in each group). One-way analysis of variance (ANOVA) followed by Tukey's post hoc tests were conducted. (B) food intake for each week (n = 6 in each group) and (C) grip strength for each week (n = 9 in each group). One-way anova followed by Fisher's protected least significant difference post hoc test were conducted at each time point. (D) Tumor weight at the end of experimental period (n = 9 in each group). Unpaired t-tests were conducted. All values are expressed as mean ± standard deviation. §P < 0.05 §§P < 0.01, significant difference vs. week 1. *P < 0.05 **P < 0.01, significant difference between C26-SED and C26-RUN. ††P < 0.01, significant difference between CON-SED and C26-SED. ##P < 0.01, significant difference between CON-SED and C26-RUN.
